# *A* *spergillus fumigatus* Hypoxia Adaptation Is Critical for the Establishment of Fungal Keratitis

**DOI:** 10.1167/iovs.65.4.31

**Published:** 2024-04-18

**Authors:** Jorge D. Lightfoot, Emily M. Adams, Manali M. Kamath, Becca L. Wells, Kevin K. Fuller

**Affiliations:** 1 Department of Ophthalmology, University of Oklahoma Health Sciences Center, Oklahoma City, Oklahoma, United States; 2 Department of Microbiology and Immunology, University of Oklahoma Health Sciences Center, Oklahoma City, Oklahoma, United States

**Keywords:** fungal keratitis (FK), hypoxia, fungal virulence

## Abstract

**Purpose:**

The poor visual outcomes associated with fungal keratitis (FK) underscore a need to identify fungal pathways that can serve as novel antifungal targets. In this report, we investigated whether hypoxia develops in the FK cornea and, by extension, if fungal hypoxia adaptation is essential for virulence in this setting.

**Methods:**

C57BL/6J mice were inoculated with *Aspergillus fumigatus* and *Fusarium solani var. petroliphilum* via topical overlay or intrastromal injection. At various time points post-inoculation (p.i.), animals were injected with pimonidazole for the detection of tissue hypoxia through immunofluorescence imaging. The *A. fumigatus srbA* gene was deleted through Cas9-mediated homologous recombination and its virulence was assessed in the topical infection model using slit-lamp microscopy and optical coherence tomography (OCT).

**Results:**

Topical inoculation with *A. fumigatus* resulted in diffuse pimonidazole staining across the epithelial and endothelial layers within 6 hours. Stromal hypoxia was evident by 48 hours p.i., which corresponded to leukocytic infiltration. Intrastromal inoculation with either *A. fumigatus* or *F. solani* similarly led to diffuse staining patterns across all corneal cell layers. The *A. fumigatus srbA* deletion mutant was unable to grow at oxygen levels below 3% in vitro, and corneas inoculated with the mutant failed to develop signs of corneal opacification, inflammation, or fungal burden.

**Conclusions:**

These results suggest that fungal antigen rapidly drives the development of corneal hypoxia, thus rendering fungal SrbA or related pathways essential for the establishment of infection. Such pathways may therefore serve as targets for novel antifungal intervention.

A*spergillus fumigatus* (*A. fumigatus*) is a ubiquitous mold and an important agent of human infection. It is the predominant fungal pathogen of the lungs, for example, where it can cause an allergic bronchopneumonia in atopic individuals or an invasive infection in those with severely compromised immune systems.^1^^,^^2^ The latter disease, termed invasive pulmonary aspergillosis (IPA), carries a mortality rate of 40% to 90% depending on the extent of immune suppression or fungal dissemination to distal organs.^3^^,^^4^ *A. fumigatus* is also an important cause of fungal keratitis (FK), a sight-threatening infection of the cornea that can affect otherwise healthy individuals that are inoculated through ocular trauma or contact lens wear.^5^^,^^6^ FK results in corneal perforation or the need for corneal transplantation in 40% to 60% of cases and long-term vision impairment in up to 70% of patients.^7^ The poor patient outcomes in either IPA or FK is due, in part, to the inadequacy of currently available antifungals.^8^^–^^10^ In principle, fungal proteins or signaling pathways that promote adaptation and growth within the host tissue could serve as novel drug targets, but the degree to which pathways differ as a function of host site is poorly understood. Accordingly, we seek to identify fungal pathways that support growth in both the lungs and corneas to facilitate the development of antifungals with dual use.

As the lung is perfused by ambient air (approximately 20% O_2_), it is perhaps unexpected that hypoxia develops during IPA. Nevertheless, Grahl et al. demonstrated that the chemical pimonidazole (Hypoxyprobe), which binds to proteins within hypoxic cells, accumulates within inflammatory foci of *A. fumigatus*-infected murine lungs several days after inoculation.^11^^–^^13^ These low oxygen microenvironments (<1% O_2_) likely arise due to several factors, including a physical reduction of oxygen diffusion through the inflamed and necrotic tissue, as well as a rapid metabolic consumption of molecular oxygen by the inflammatory cells themselves.^14^^,^^15^ Ethanol, which is not produced by host cells, is furthermore detectable in infected lungs at time points corresponding to the positive pimonidazole staining, indicating a shift in fungal metabolism toward fermentation.^13^ Taken together, the cell compositional, structural, and metabolic changes that occur within the lungs over the course of *A. fumigatus* infection cumulatively result in an oxygen depletion to which the fungus must adapt.^13^^,^^16^^,^^17^

A critical component of the *A. fumigatus* hypoxic response is the regulation of membrane sterol metabolism.^18^^–^^20^ Like most fungi, *A. fumigatus* relies on the *de novo* synthesis of ergosterol to maintain membrane homeostasis and growth. The synthesis of each molecule of ergosterol is energetically costly, however, requiring a large amount of ATP equivalents and 12 molecules of oxygen.^21^^,^^22^ This is significant in two related ways, the first being that as molecular oxygen becomes limiting, so too will the concentration of membrane ergosterol. Second, fungi lack an apparent ortholog of the mammalian hypoxia-inducible factor proteins (Hif-1α and HIF-2α) and cannot sense environmental oxygen directly.^22^ Thus, the depletion of membrane sterols serves as proxy for hypoxia and triggers the adaptive response, which in *A. fumigatus* is chiefly driven by Zn-finger transcription factor SrbA (for sterol-regulatory element-binding protein).^20^ Upon sterol depletion, SrbA is cleaved by regulatory proteins, translocates from the endoplasmic reticulum (ER) membrane to the nucleus and, as the name suggests, drives the expression of several genes within the ergosterol biosynthetic pathway.^19^^,^^23^^,^^24^ Whereas wild-type (WT) *A. fumigatus* grows almost uninhibited at oxygen concentrations as low as 0.2%, the *srbA* deletion mutant (∆*srbA*) is growth ablated at oxygen concentrations at or below 3%.^25^ The ∆*srbA* mutant is furthermore avirulent in multiple models of IPA, thus underscoring the importance of fungal hypoxia adaptation within the lungs.^20^

It is currently unknown whether hypoxia develops in the fungal-infected cornea. On one hand, the primary site of fungal growth during FK, the central stroma, is separated from ambient air by a thin epithelium and a tear film that altogether measure just 550 µM in thickness in humans.^26^ Thus, it may be expected that the cornea is unique among other host tissues in that it, and the infecting fungus, remain well-perfused by atmospheric oxygen during infection. On the other hand, the cornea lacks a vascular supply of oxygen and massive leukocytic infiltration and tissue edema are hallmarks of FK pathology.^27^ These factors may therefore lead to oxygen depletion in the central cornea due to an increased diffusion barrier of atmospheric O_2_ (tissue thickness) and its conversion to reactive oxygen species (ROS) by host inflammatory cells, altogether mirroring the pathobiology of pulmonary aspergillosis.^28^^–^^31^ In this report, we demonstrate that inoculation of murine corneas with *A. fumigatus* does indeed lead to the development of hypoxia, not just in the central cornea, but across all tissue layers including the epithelium. Interestingly, and unlike the lungs, this hypoxia precedes the infiltration of peripheral leukocytes, suggesting that fungal antigen can influence oxygen metabolism in corneal resident cells. Importantly, we demonstrate that an *A. fumigatus srbA* deletant is unable to establish infection in our murine model, suggesting that the pathway could serve as a novel target for the treatment of FK.

## Materials and Methods

### Strains and Culture Conditions

Strains and plasmids used in this study are listed in Supplementary Table S1. All *A. fumigatus* strains were derived from Af293 and maintained on glucose minimal medium (GMM; adapted from Cove 1966), containing 1% (w/v) glucose and 10 µM ammonium tartrate as a nitrogen source; pH = 6.5.^32^ Gelatin minimal media was prepared in the same way, substituting 1% (w/v) of gelatin (gelatin, type B: from bovine skin; Sigma, St. Louis, MO, USA). Normoxic conditions consisted of ambient oxygen (19–21%) with 5% CO_2_ and hypoxic conditions consisted of 1% O_2_, 5% CO_2_, and N_2_ balance. All incubations were performed at 35°C. *F. solani* was maintained on potato dextrose agar and incubated at 30°C.

### Generation of Fungal Mutants

The *A.* *fumigatus* strain Af293, a clinical isolate from a patient with IPA, was used as the background strain.^33^ The mCherry fluorescent genes was introduced into this background and the resulting strain (Af293 *PgpdA-mCherry-hph*) was used as the WT in this study. Targeted gene deletions were performed as described in Al Abdallah et al. 2019 and Szewczyk et al. 2006.^34^^,^^35^ Briefly, protoplasts were generated from germlings using the following enzymes for 3.5 hours: 5 mg/mL of lysing enzymes from *Trichoderma harzanium*, 5 mg/mL of Driselase, and 100 µg of chitinase in OSM (1.2 M MgSO_4_, 10 mM sodium phosphate buffer pH 5.8) at 30°C. Protoplasts were then purified using a density-based trapping buffer (0.6 M sorbitol, 0.1 M Tris-HCl) layered over the lysing enzyme buffer and spun at 5000 × g for 15 minutes at 4°C. The protoplasts were pipetted from the interphase, washed via centrifugation for 5 minutes at 5000 × g, in STC (1.2 M sorbitol, 10 mM CaCl_2_, 10 mM Tris-HCL pH 7.5), enumerated via hemocytometer, and diluted to 5 × 10^5^ protoplasts/mL.

CRISPR RNAs (cRNAs) and primers used in this study are listed in Supplementary Table S2. The crRNAs were designed to flank the coding region of the *srbA* gene (Afu2g01260) and conjugated to tracrRNAs (IDT) by combining equimolar amounts with nuclease free duplex buffer (IDT). Then, 3.33 µL of each reagent is combined and incubated at 95°C for 5 minutes before cooling them to room temperature. The crRNAs were then conjugated to 1 µg of Alt R Cas9 (IDT) in 10 µL of Cas9 working buffer (20 mM HEPES, 150 mM KCl pH of 7.5) at 37°C for 30 minutes. Then, 200 µL of the protoplasts were transferred to a fresh Eppendorf tube and mixed with 10 µg of the phleomycin repair template (amplified from p402R), flanked with 35bp regions of microhomology directly adjacent to the Cas9 mediated double stranded breaks. The Cas9 conjugated gRNAs were combined and introduced to the protoplasts. Then, 50 µL of polyethylene glycol (PEG) solution (60% PEG M.W. 3350 [w/v], 50 mM CaCl_2_, 50 mM Tris-HCL pH 7.5) were added to the protoplast/DNA mixture and mixed by flicking the tube. The mixture was then placed on ice for 50 minutes. An additional 500 µL of the PEG solution was added and incubated at room temperature for 30 min. The PEG solution was removed by centrifuging the protoplasts at >13,000 revolutions per minute (rpm) for 3 minutes and pipetting off the supernatant. The protoplasts were centrifuged at >13,000 for an additional 1 minute to remove the remaining PEG solution before they were resuspended in 1 mL of STC. Then, 100 µL of this suspension was plated onto 20 mL of GMM + 1.2 M sorbitol and incubated at room temperature overnight. A 10 mL overlay of top agar (GMM with 5 g/L of agar) and 375 µg/mL phleomycin was poured onto the protoplasts. These plates were placed at 35°C and monitored for the formation of microcolonies for 48 hours. Colonies that grew under selection were screened via PCR for a replacement of the *srbA* coding region with the phleomycin resistance marker *bleR*.

The *srbA* locus was complemented into the *atf4* locus using a single crRNA mediated cut site and a marker-less transformation strategy.^36^ Protoplasts were recovered in hypoxia (1% O_2_, and 5% CO_2_) after an overnight incubation at room temperature and ambient oxygen conditions. Colonies that were able to grow in hypoxia were then screened via PCR for the correct insertion of the *srbA* locus into the *atf4* locus.

### Topical Murine Keratitis Model

All animal experiments were performed according to the Association for Research in Vision and Ophthalmology guidelines for the use of animals in vision research and approved by the University of Oklahoma Health Sciences Center Institutional Animal Care and Use Committee (Protocol: 20-060-CI). C57BL/6J mice, male or female as indicated in the figure caption, were obtained from Jackson Laboratories (Bar Harbor, ME, USA). On the day preceding fungal infection, the animals were immunosuppressed via intraperitoneal (i.p.) injection with 100 mg/kg of methylprednisolone. On the day of infection, conidia were swollen in YPD (10% yeast extract, 20% peptone, and 20% glucose) for 4.5 hours at 35°C to induce isotropic growth (swelling) and metabolic activation. The inoculum was normalized to an OD of 0.8 at 360 nm. For the topical inoculation procedure, mice were anesthetized by i.p. injection of ketamine (100 mg/kg) and xylazine (6.6 mg/kg) and the center of the right cornea was ulcerated with an Algerbrush to remove the central epithelium to a diameter of approximately 1 mm. Then, 5 µL of the inoculum were overlaid onto the ulcerated cornea and allowed to rest on the ocular surface for 20 minutes before being wicked off with a Kim wipe. Buprenorphine SR (1 mg/kg) was administered subcutaneously intra-operatively for analgesia.

For the experiment involving killed inoculum, half of the inoculum (described above) was killed by incubation in 70% ethanol for 20 minutes and then washing twice in PBS before being normalized to an OD of 0.8 at 360 nm. Conidia killing was confirmed by plating an aliquot onto inhibitory mold agar and observing no CFUs. Live conidia were subjected to the same washing and normalization steps. Buprenorphine SR (1 mg/kg) was administered subcutaneously intra-operatively for analgesia.

### Intrastromal Murine Keratitis Model

Infections were performed as described in Leal et al. 2014.^28^ Briefly, male C57BL/6J mice were immunosuppressed with 100 mg/kg of methylprednisolone on the day preceding infection. On the day of infection, the mice were anesthetized with ketamine and xylazine as described above. A small pocket was introduced into the corneal epithelium with a 20.5-gauge needle before a glass-pulled capillary needle was inserted into the opening. Using a programmable pneumatic microinjection system (Microdata Instruments, Plainfield, NJ, USA) 2 µL of 2.5 × 10^7^ conidia/mL were injected into the corneal stroma. Buprenorphine SR was administered subcutaneously intra-operatively for analgesia.

### CFU Analysis

Corneas were resected and placed in 2 mg/mL of Collagenase I (Sigma SCR103) in PBS within a 1.5 mL Eppendorf tube. The tubes were oscillated at 50 Hz in two 30 second pulses in a Qiagen TissueLyzer before they were incubated at 37°C for 15 minutes. The homogenate was then pipetted vigorously 8 times before being returned to 37°C for an additional 15 minutes. These steps were repeated once more before the homogenate was diluted 1:1 in PBS and 100 µL was plated onto inhibitory mold agar (IMA). Plates were incubated overnight at 35°C before colonies were enumerated.

### Micron IV Imaging and Analysis

Mice were anesthetized with isoflurane and slit lamp images were taken using a Micron IV Biomicroscope (Phoenix Technology Group, Pleasanton, CA, USA). Bright field images were captured at a 66 ms exposure. These images were randomized and scored by two blinded reviewers using previously established criteria.^37^ Briefly, the images were scored on a scale of 0 to 4 based on surface regularity, area of opacification, and density of opacification. The scores were averaged for each individual cornea and statistical analysis was performed on the experimental group.

### Optical Coherence Tomography Imaging and Analysis

Corneas were imaged using the Bioptigen spectral-domain optical coherence tomography (OCT) system (Leica Microsystems, Buffalo Grove, IL, USA) to measure corneal edema and inflammation. Mice were anesthetized with isoflurane and a 4 × 4 mm image is scanned with the 12 mm telecentric lens. Reference arm calibration was completed by the manufacturer and set to 885.

Corneal scans were digitally overlaid with an 11 × 11 Spider plot, and the 13 central points were marked at the endothelium and the epithelium. The differences between these points were calculated as the corneal thickness in millimeters. These 13 measurements were then averaged and plotted as a single data point.

### Hypoxyprobe Immunohistochemical Staining

Mice inoculated either through the topical or intrastromal route were injected i.p. with 60 mg/kg of Hypoxyprobe (Hypoxyprobe, Inc. Burlington, MA, USA) dissolved in PBS, and 90 minutes before they were euthanized by intraperitoneal injection of ketamine (200 mg/kg) and xylazine (13.2 mg/kg). Eyes were enucleated and fixed in paraformaldehyde. To deparaffinize the sections, the slides were warmed up in a 40°C oven for 20 minutes. The slides were then placed in a xylene bath for 5 minutes 3 times. Sections were rehydrated in 3 separate 95% ethanol baths 3 times before being washed in washing buffer (0.2% [w/v] Brij 35 in PBS). Slides were incubated at 90°C for 20 minutes in High pH IHC Antigen Retrieval Solution (Thermo Fisher Scientific Catalog Number 00-4956) and allowed to cool to room temperature. Slides were then washed and blocked with Cas-Block (Life Technologies 00-8120) for 120 minutes. Sections were incubated with the primary antibody, 1:100 FITC-Mab1 (Hypoxyprobe mouse MAb clone 4.3.11.3) overnight at room temperature. Slides were then washed and incubated for 60 minutes at room temperature with the secondary antibody: 1:100 dilution of DyLight 594 conjugated mouse anti-FITC (Jackson Immuno Research 200-512-037). Slides were washed and dried by blotting before being mounted and counterstained using ProLong Gold Antifade Mountant with DAPI (Thermo Fisher P36931).

Imaging was performed on a Nikon E800 epifluorescent microscope. Single color channel images were captured in DAPI (exposure 3 seconds) and Dylight 594 (exposure 5 seconds). Images were processed in Metamorph (Molecular Devices, San Jose, CA, USA) to subtract the background fluorescence signal in each single channel using isotype controls as a reference. Following background normalization, all images were compiled into a single Adobe Photoshop document (version 24.7.1) and the brightness was adjusted equally across all panels to improve image visibility.

### Quantitative RT-PCR Analysis

Animals were euthanized by intraperitoneal injection of ketamine (200 mg/kg) and xylazine (13.2 mg/kg) at 24 hours post infection, and individual corneas were resected and transferred to RLT Buffer (RNeasy Mini kit; Qiagen, Germantown, MD, USA). Corneas were homogenized with a TissueLyser LT (Qiagen, Germantown, MD, USA) at 50 oscillations/second for two 30-second cycles with the Green Bead Lysis Kit (Next Advance, Troy, NY, USA). RNA was extracted from the tissue homogenate using the RNeasy Mini kit (Qiagen, USA). RNA was DNase treated using the DNase 1 kit (Millipore Sigma, Burlington, MA, USA). The RNA was quantified with the Nanodrop 2000 (Thermo Fisher Scientific, Franklin, MA, USA) and normalized for conversion to cDNA with the Protoscript II First Strand Synthesis Kit (New England Bio Labs, Beverly, MA, USA). The quantitative RT-PCR (qRT-PCR) was performed using Luna Universal qPCR SYBR green master mix (New England Bio Labs, Beverly, MA, USA) on the QuantStudio3 (Thermo Fisher Scientific, Franklin, MA, USA). Analysis was performed using QuantStudio Design and Analysis Software version 1.5.2 using the 2^(-ΔΔCt) method to measure fold change expression relative to actin. Statistics were performed on the ΔCt measurements. All primers used in this study are described in Supplementary Table S1.

### Statistical Analysis

Statistical analysis was performed on GraphPad Prism version 10.0.2. and the specific statistical tests applied for each experiment are described in the corresponding figure legends.

## Results

### Corneal Hypoxia Develops in a Topical Inoculation Model of *A. fumigatus* Keratitis

**Figure 1.**
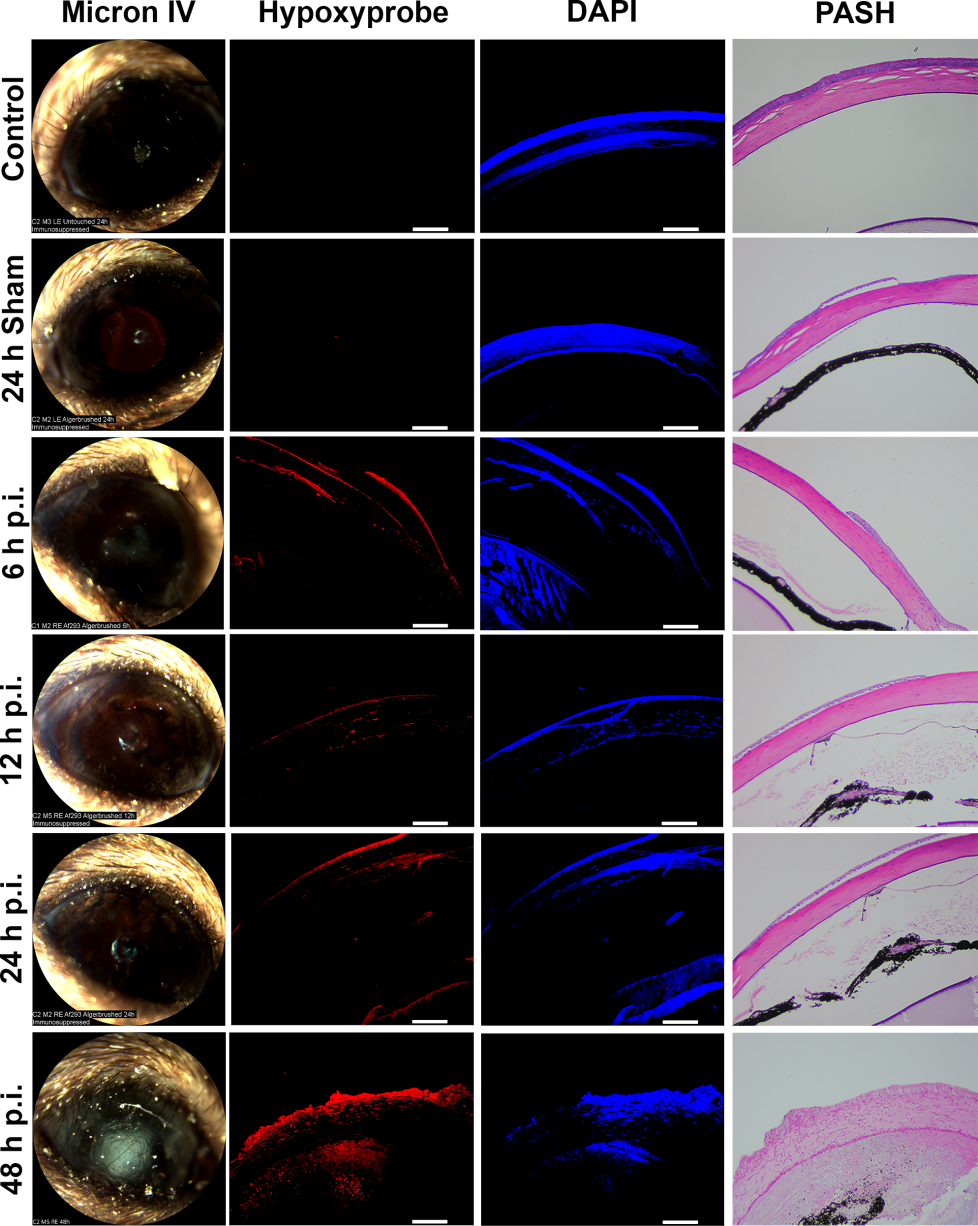
**Corneal hypoxia develops in a topical inoculation model of *A. fumigatus* keratitis.** Hypoxyprobe was injected intraperitoneally 90 minutes before harvesting eyes infected with *A. fumigatus* at the indicated time points (*n* = 2 per group). Control and sham-infected animals were similarly injected with Hypoxyprobe and taken at 24 hours p.i. Ocular sections were stained with DAPI as a control (*blue*) along with anti-Hypoxyprobe antibodies to detect Hypoxyprobe bound to proteins (*red*). Isotype controls demonstrating stain specificity can be observed in Supplementary Figure S1. A serial section was stained with periodic acid Schiff and hematoxylin (PASH) as a reference and to visualize *A. fumigatus* hyphae (*fuchsia*). Scale bars are 100 µm. All animals in this experiment were male.

To determine if *A. fumigatus* infection promotes the development of corneal hypoxia, we first used a topical inoculation model of FK, as previously described.^38^^–^^41^ Briefly, 6 to 8-week-old C57BL/6J mice were immunosuppressed with methylprednisolone 24 hours prior to infection. On the day of inoculation, an approximately 1 mm area of the central corneal epithelium was debrided with an Algerbrush and topically overlaid with *A. fumigatus* conidia that were pre-germinated (swollen, but not polarized) in nutrient-rich broth. At 6, 12, 24, and 48 hours post-inoculation (p.i.), animals were administered Hypoxyprobe by intraperitoneal injection and euthanized 90 minutes later. Serial ocular sections were either stained with Periodic acid-Schiff and hematoxylin (PASH) for histological analysis, with DAPI along anti-Hypoxyprobe antibodies for the detection of hypoxic microenvironments via immunofluorescence (IF). Whereas sham-inoculated and untouched (control) corneas were negative for Hypoxyprobe staining at all tested time points, those inoculated with *A. fumigatus* displayed positive staining in the epithelium, endothelium, and even discrete layers of the stromal collagen beginning at the earliest tested time point (6 hours p.i.). The central epithelial ulcer generated for the inoculation typically reforms within 24 hours in un-inoculated corneas, but does not reform in infected corneas; consequently, the sections shown in Figure 1 are peripheral to the original ulcer (i.e. closer to the limbus). The distribution and intensity of the Hypoxyprobe staining was markedly enhanced at 48 hours p.i., which corresponded to both the presence of leukocytic infiltration and tissue damage upon evaluation of PASH stained sections as well as the onset of corneal opacification upon slit-lamp examination (see Fig. 1).

**Figure 2.**
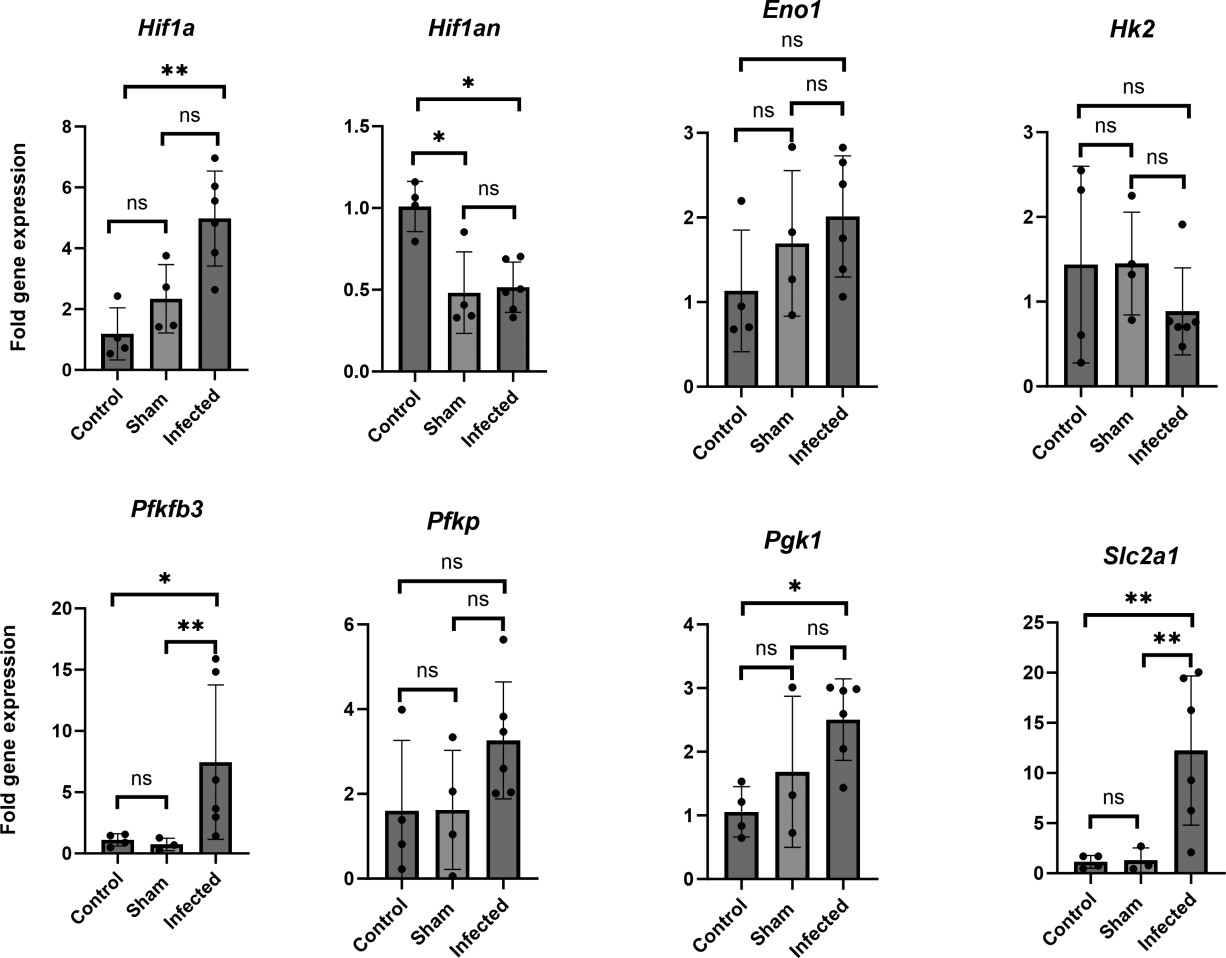
**Hif1α and glycolytic gene expression is elevated in *A. fumigatus* infected corneas.** *A. fumigatus* (*n* = 6) or sham-infected corneas (*n* = 4) were collected at 24 hours p.i. along with untouched corneas as controls (*n* = 4). Following RNA isolation and first-strand cDNA synthesis, qRT-PCR was used to measure relative expression of the indicated genes with actin serving as a reference to plot the relative fold change 2^(-ΔΔCT); error bars reflect the standard deviation of the mean. For statistical analysis, the average ∆Cts of the groups were analyzed in a One-way ANOVA *P < 0.05, **P < 0.01. All animals in this experiment were male.

To determine if the hypoxia detected in our model was sufficient to impact the physiology of corneal resident cells, *A. fumigatus* or sham-inoculated corneas were collected 24 hours p.i. and the expression of known hypoxia-regulated genes were analyzed by qRT-PCR. Among the statistically elevated genes, we included the master hypoxia regulator, Hif-1α, the glycolytic enzyme 6-phosphofructo-2kinase/fructose-2,6-bisphosphatase 3, Pfkfb3, the tricarboxylic acid cycle enzyme phosphoglycerate kinase, Pgk1, and the glucose transporter, Slc2A1 (Fig. 2). The upregulation of these genes is consistent in herpes simplex virus-1 (HSV-1) infected corneas, which also stain positively with pimonidazole, and suggests a shift in host cell metabolism toward glycolytic metabolism and fermentation.^42^ Conversely, the gene encoding the Hif-1α inhibitor (Hif1an) was statistically downregulated in the FK corneas (see Fig. 2), which is consistent with the activation of Hif-1α signaling.^43^ Taken together, the IF and gene expression data support a model in which *A. fumigatus* infection leads to rapid oxygen depletion and altered host cell metabolism across the cornea in a manner that precedes, but is exacerbated by, the influx of inflammatory cells into the tissue.

### Hypoxia Development Is Chiefly Driven by the Host Response to Fungal Antigen

**Figure 3.**
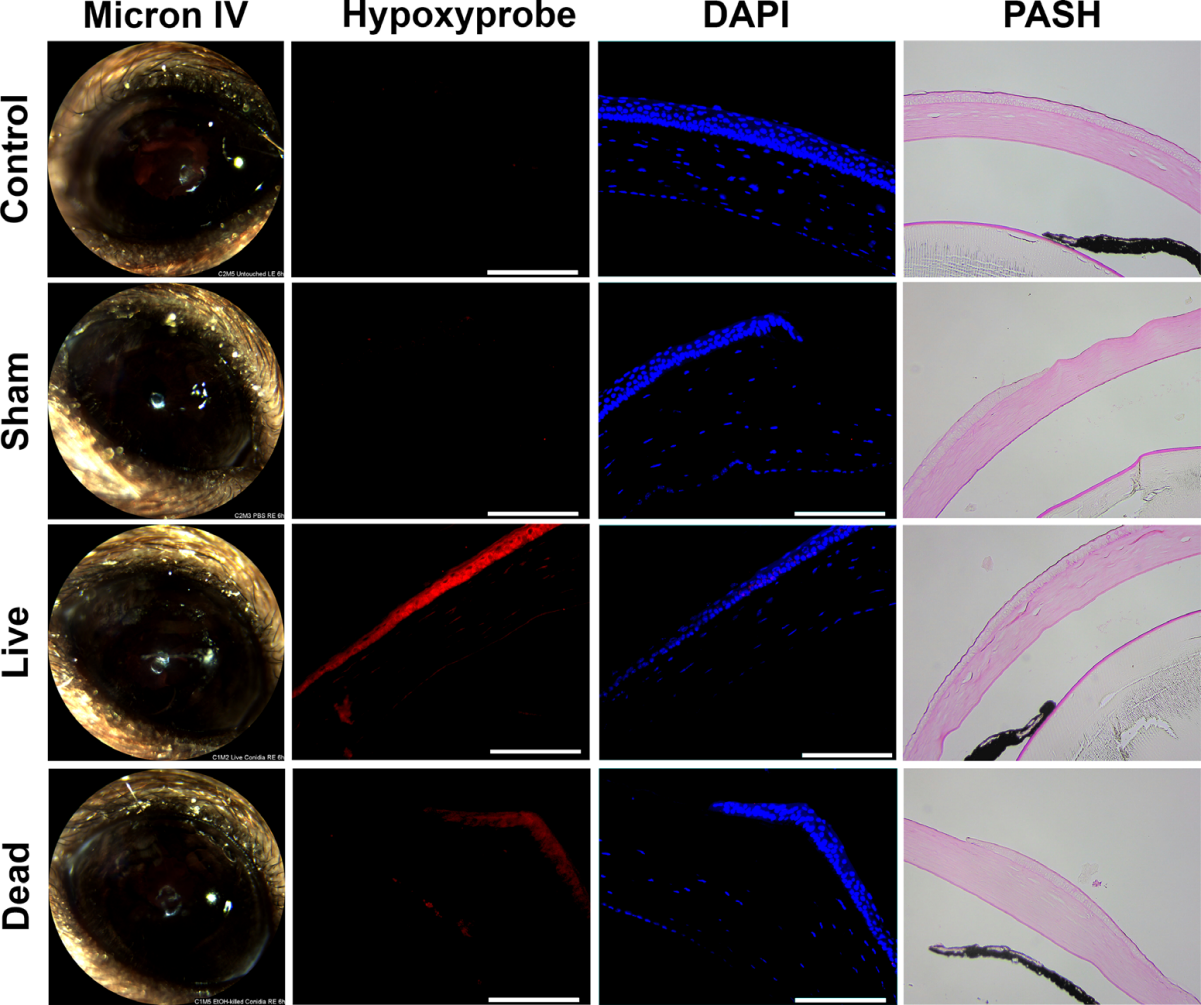
**Hypoxia development is driven primarily by the host response to fungal antigen.** *A. fumigatus* conidia were swollen in YPD and half of the inoculum was killed with 70% ethanol. Corneas were ulcerated and overlaid with either the live conidia (*n* = 3), killed conidia (*n* = 4), PBS as a sham (*n* = 3), or left untouched (*n* = 3). Hypoxyprobe was injected intraperitoneally 90 minutes prior to euthanization and harvesting of eyes at 6 hours p.i. Ocular sections were stained with DAPI as a control (*blue*) along with anti-Hypoxyprobe antibodies to detect Hypoxyprobe bound to proteins (*red*). A serial section was stained with periodic acid Schiff and hematoxylin (PASH) as a reference and to visualize *A. fumigatus* hyphae (*fuchsia*). Scale bars are 100 µm. All animals in this experiment were male.

The inoculation of metabolically active fungus onto the corneal surface in the above study could drive hypoxia across the epithelium in at least two, but possibly additive ways: first, the direct interaction of fungal antigen with epithelial cells leads to pro-inflammatory signaling and increased oxygen consumption by those cells; and second, the fungus itself may consume and locally deplete oxygen availability to the tissue layer. To distinguish between these possibilities, we repeated the Hypoxyprobe staining experiment with the inclusion of killed *A. fumigatus* inoculum to determine the influence of fungal antigen alone. As shown in Figure 3, and in contrast to sham-inoculated or untouched control eyes, both the live and dead fungal inocula resulted in the development of a positive Hypoxyprobe signal at 6 hours p.i. Interestingly, the intensity of the staining appeared stronger in tissues inoculated with live conidia, suggesting that whereas fungal antigen is sufficient to drive the development of hypoxia in our model, an exacerbating effect of oxygen depletion by the fungus cannot be ruled out.

### Hypoxia Development Is Shared Across Multiple Models of FK

**Figure 4.**
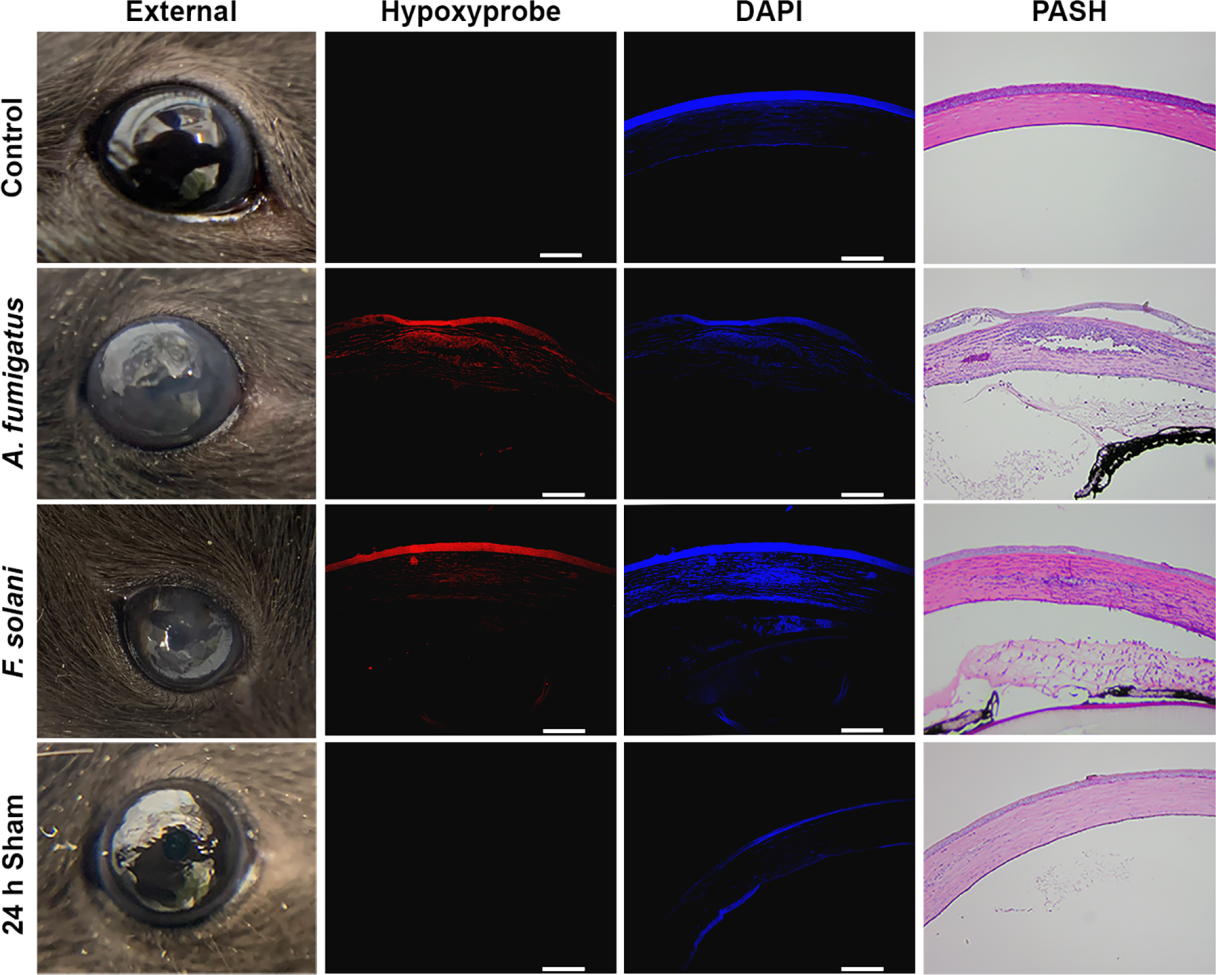
**Hypoxia development is shared across multiple models of FK.** Corneas were injected intrastromally with either *A. fumigatus* conidia, *F. solani var. petroliphilum* conidia, PBS, or left untouched (*n* = 3/group). Hypoxyprobe was injected intraperitoneally 90 minutes prior to euthanization and harvesting of eyes at 6 hours p.i. Ocular sections were stained with DAPI as a control (*blue*) along with anti-Hypoxyprobe antibodies to detect Hypoxyprobe bound to proteins (*red*). A serial section was stained with periodic acid Schiff and hematoxylin (PASH) as a reference and to visualize fungal hyphae (*fuchsia*). Scale bars are 100 µm. All animals in this experiment were male.

To determine if the development of corneal hypoxia is relevant to other murine models of FK, we next evaluated patterns of Hypoxyprobe staining within an intrastromal injection model whereby fungal conidia were delivered directly into the underlying stroma through a small needle puncture.^44^^–^^46^ In addition to *A. fumigatus*, we also performed this method with another predominant FK pathogen, *Fusarium solani var. petroliphilum*, to assess whether hypoxia development is pathogen-specific.^47^^–^^49^ In contrast to the technical ease and high infection rate associated with topical inoculation, we experienced a high failure rate (approximately 50%) for the intrastromal approach that that was not obvious until animals did or did not develop signs of disease at around 24 hours p.i. As such, early (subclinical) time points could not be confidently analyzed as they were with the topical infection model. Nevertheless, animals that did develop signs of corneal opacification at 24 hours p.i. displayed not only a positive Hypoxyprobe signal in the intrastromal space as expected, but also a strong signal across the entire corneal epithelial and endothelial layers similar to what was observed following topical inoculation (Fig. 4). Unstained infected tissue sections or those stained with isotype control antibodies did not display a positive fluorescent signal (Supplementary Fig. S1). Taken together, hypoxia development across the corneal cell layers was dependent neither on the mode of fungal inoculation nor the pathogen evaluated in this study.

### The SrbA Transcription Factor Is Required for Growth of *A. fumigatus* Under Hypoxia, but not on Proteinaceous Substrates

**Figure 5.**
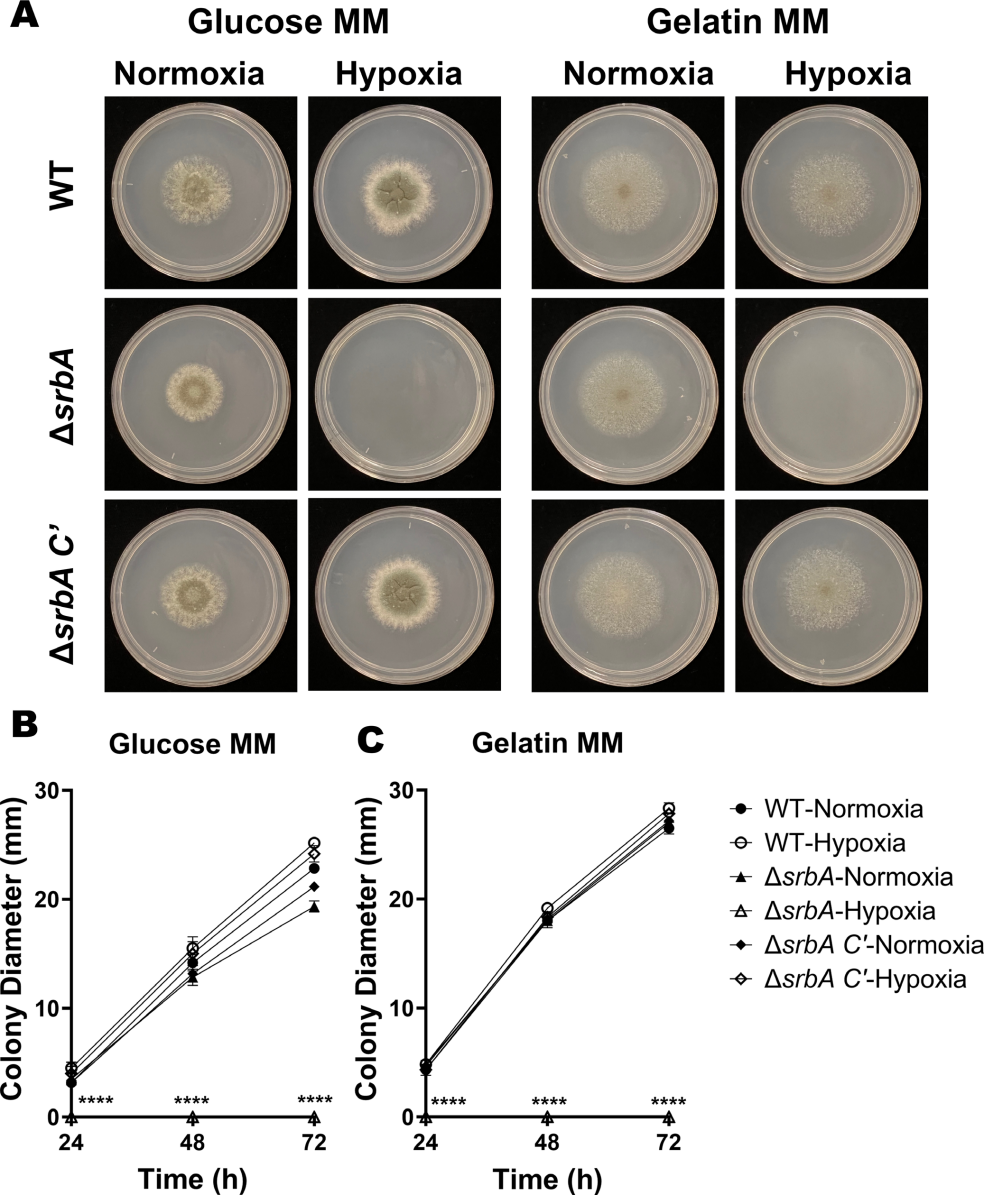
**The SrbA transcription factor is required for growth of *A. fumigatus* under hypoxia, but not on proteinaceous substrates.** (**A**) Conidia of wild-type (WT) *A. fumigatus*, the *srbA* knockout (∆*srbA*), or complemented mutant (∆*srbA* C′) were inoculated onto glucose or gelatin minimal media and photographed at 72 hours following incubation in either normoxia (19-21% O_2_) or hypoxia (1% O_2_) at 35°C. (**B,** **C**) The mean colony diameters each day (*n* = 3/group) were compared by the Kruskal-Wallis test: **P ≤ 0.01, ****P ≤ 0.0001.

We next wanted to determine if the hypoxia that develops during FK has an influence on the pathobiology of the fungus. Toward this end, the gene encoding the *srbA* transcription factor was replaced with the phleomycin resistance cassette in our mCherry expressing *A. fumigatus* strain (strain Af293 *PgpdA-mCherry-hph*) using Cas9-mediated homologous recombination. Subsequent generation of the complemented strain (*∆srbA* C’) was achieved through the targeted integration of the wild-type *srbA* allele into the *atf4* locus of the *∆srbA* mutant (Supplementary Fig. S2).^36^ The isogenic strains were indistinguishable on GMM, containing glucose and ammonium, under atmospheric conditions of 21% O_2_ (normoxia). By contrast, *∆srbA* was growth ablated on GMM at O_2_ concentrations below 3%, thus confirming the essential role for SrbA in *A. fumigatus* hypoxia adaptation previously described (Fig. 5).

Given that the cornea is rich in alternative macronutrients, primarily collagen and glycosaminoglycans that comprise the stromal extracellular matrix, we further tested the growth of the strains on media containing gelatin (collagen hydrolysate) as the sole carbon and nitrogen source. As shown in Figure 3, all strains were indistinguishable with respect to colony morphology and linear growth rate in normoxia. Similar to glucose, however, *∆srbA* was growth ablated in hypoxia on this medium (see Fig. 5). We reasoned, therefore, that any observed virulence defect of *∆srbA* could be attributed to the influence of oxygen depletion in the tissue, rather than some previously uncharacterized role for SrbA in the metabolism of cornea-relevant macronutrients.

### *A. fumigatus* SrbA Is Essential for the Establishment of Fungal Keratitis

**Figure 6.**
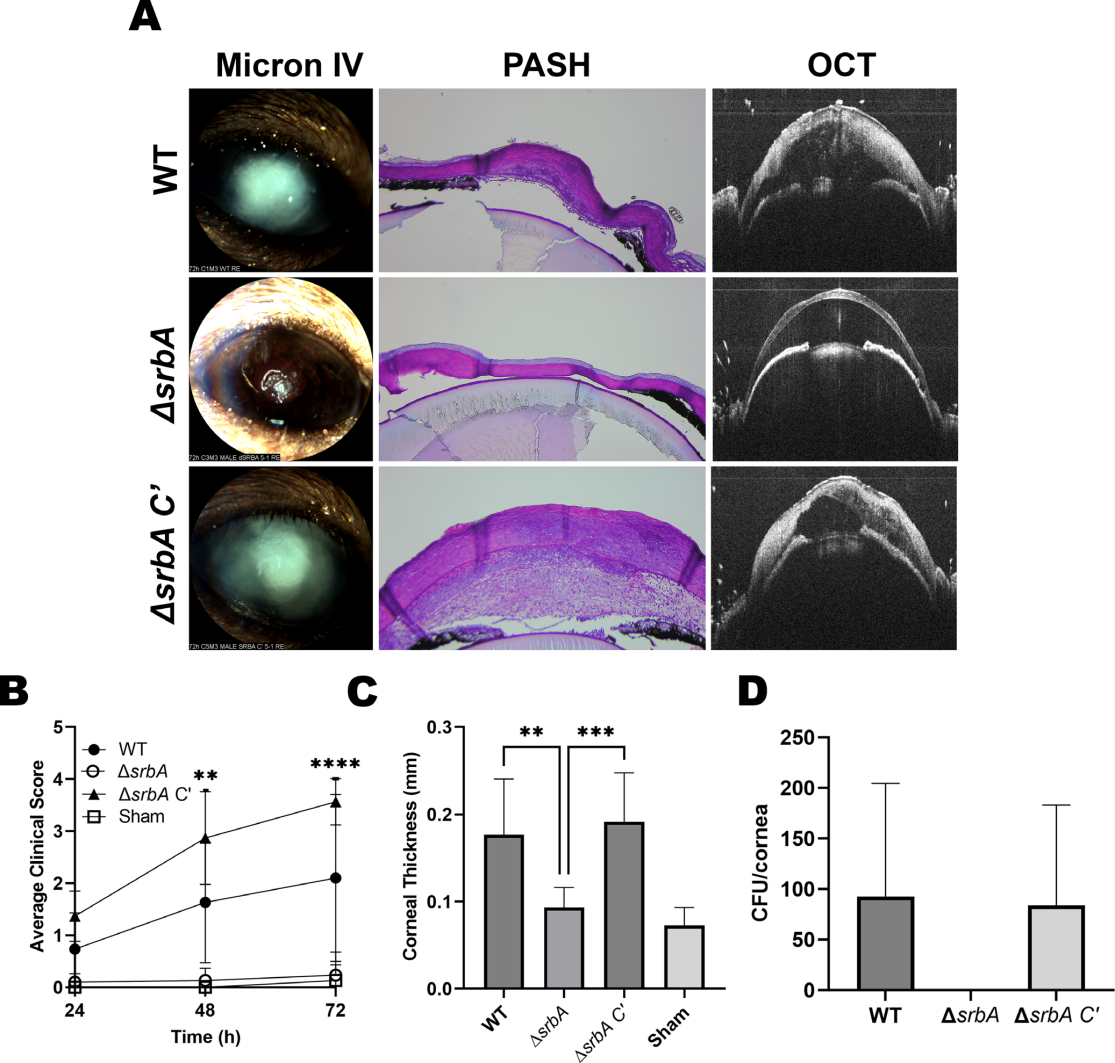
***A. fumigatus* SrbA is essential for the establishment of fungal keratitis.** (**A**, **B**) Corneas were ulcerated and overlaid with inocula of the indicated strains and monitored daily with a slit-lamp for clinical disease scoring (*n* = 5 males, 5 females/group). (**A**, **C**) At 72 hours p.i., the corneas were also evaluated by optical coherence tomography (OCT) for corneal thickness measurement (*n* = 5 males, 5 females /group). (**A**, **D**) At 72 hours, animals were euthanized and their eyes were either processed for histology (PASH staining) or the corneas were resected and homogenized for colony forming unit quantitation (WT, *n* = 1 male, 2 females; ∆*srbA* = 2 male, 2 females; ∆*srbA C*′, *n* = 5, 3 males, 2 females). Groups in all panels were compared by One-way ANOVA: **P ≤ 0.01, ***P ≤ 0.001, ****P ≤ 0.0001.

The relative capacity of the strains to infect and drive disease was next evaluated in the above-described topical model of FK. In contrast to the WT and *srbA C’* groups, animals inoculated with ∆*srbA* failed to demonstrate any signs of disease upon external (slit-lamp) evaluation (Figs. 6A, 6B). Alterations in corneal structure were evaluated more thoroughly with OCT, which provides a cross-sectional image of the anterior segment in live animals that can then be used for a quantitative assessment of corneal thickness. As expected, OCT revealed thickened corneal tissue and refraction in WT and *srbA C’*-infected corneas, which was indicative of the corneal edema and inflammation that are characteristic of FK. By contrast, and consistent with the external images, corneas inoculated with *∆srbA* were indistinguishable from sham-inoculated controls (Fig. 6C). Tissue sections taken at 72 hours p.i. were consistent with the OCT findings and demonstrated that WT and *srbA C’*-infected corneas were marked by ulcerated and structurally abnormal corneas, with massive immune cell infiltration in both the cornea and/or anterior chamber. Fungal hyphae were also observed through the depth of the cornea in these two groups on histology, and colony forming unit (CFU) assessment from homogenized corneas indicated comparable fungal loads. Histology of *∆srbA*-infected corneas, on the other hand, displayed normal epithelial and stromal architecture, minimal leukocytic infiltration, and had no visible fungal growth, the latter which was confirmed upon CFU analysis (Fig. 6D). These results, which support a critical role of *A. fumigatus* SrbA in the establishment of corneal infection, were replicated in an independent experiment using male animals (Supplementary Fig. S3).

## Discussion

Despite its proximity to ambient air, the cornea can become hypoxic downstream to pathologies that increase oxygen consumption by corneal cells or decrease oxygen diffusion through the tissue, such as chemical burn or edema.^50^^–^^54^ In this study, we demonstrated that oxygen depletion, as assessed through pimonidazole (Hypoxyprobe) staining, is also a prominent feature of two non-contact lens models of *Aspergillus* keratitis in mice. The evaluation of our sham-infected corneas suggests that the formation of hypoxia is not related to the predisposing ocular trauma that that precedes inoculation (e.g. debridement or needle puncture), but is likely dependent on the host response to fungal antigen. The potential mechanisms, consequence, and therapeutic implications of these findings will be discussed below.

Rao and Suvas, similarly used Hypoxyprobe staining to demonstrate that mouse corneal epithelial cells become hypoxic 4 days following inoculation with HSV-1.^42^ The authors further demonstrate that antibody-mediated neutrophil depletion ablates hypoxia formation, indicating that oxidative metabolism by the inflammatory cells is an underlying cause of oxygen depletion in the model. In our study, we similarly noted an enhanced distribution of Hypoxybrobe staining in FK corneas at time points that correspond with immune cell infiltration (beginning at 48 hours p.i.). Notably, however, diffuse staining was also observed across the epithelium and endothelial layers at times that precede clinical or histological signs of inflammation (6 hours p.i.). It is known that corneal epithelial cells can respond directly to fungal antigen via Dectin-1 and TLR signaling pathways, leading to NF-kB activation and the secretion of pro-inflammatory cytokines and chemokines.^55^^–^^60^ It has furthermore been described in other cell types (e.g. adipocytes, retinal pigmented epithelial cells, and macrophages) that mitochondrial activity and ROS production are increased shortly after exogenous treatment with such cytokines.^59^^,^^61^^,^^62^ Thus, we propose that a direct response of epithelial cells to the fungus in our topical inoculation model leads to autologous and paracrine signaling that promotes rapid oxygen depletion across the tissue. When the direct fungal-epithelial interaction is largely bypassed, such as in our intrastromal injection model, we predict that stromal cells (e.g. macrophages, dendritic cells, and even keratocytes/fibroblasts) initiate the cytokine release that then diffuses to both the epithelial and endothelial layers.^38^^,^^59^^,^^63^ Future directions will be focused on identifying the relevant corneal cell populations and molecular signaling events that promote tissue hypoxia in response to fungal antigen.

Numerous studies have shown that the function of all corneal cell types can be altered in low oxygen environments.^64^^,^^65^ We therefore propose that corneal hypoxia is not only a consequence of FK pathology, but also drives key features of the disease. In our topical model, for example, we observed that the epithelial ulcer fails to reform in infected corneas, whereas it does so by 24 hours in sham-inoculated controls (see Fig. 1 and data not shown). Although direct fungal-mediated damage to the epithelial cells cannot be ruled out as a primary cause of this phenomenon, previous studies have shown that hypoxia decreases the proliferation rate of the basal epithelial cell layer and causes delay in epithelial wound healing.^66^^,^^67^ Several mechanisms may account for this, including the hypoxia-mediated activation of the polo-like-kinase 3 (Plk3) leading to cell-cycle arrest, as well as a disruption of Ca^2+^ signaling from corneal nerves to epithelial cells.^52^^,^^53^ As epithelial ulcers also develop in later stages of our intrastromal model as well as in patients with FK, we suggest this may be initiated or exacerbated by the influence of hypoxia on epithelial cell biology.^68^^,^^69^ Low oxygen may also impact important inflammatory signaling events in the epithelium that influence fungal clearance. Leal et al., for example, demonstrated that (1) TLR4 deficiency results in increased fungal (*A. fumigatus*) burden relative to infected wild-type controls, and (2) TLR4^−/−^ mice do not display defects in neutrophil recruitment during infection, suggesting that the TLR4 pathway has a specific role in promoting the fungicidal activity of these cells once they are recruited to the cornea.^59^ Importantly, Hara and colleagues showed that human corneal epithelial cells cultured under hypoxic conditions display reduced TLR4 expression.^60^ Thus, the development of hypoxia during FK, which we propose is principally driven by the pro-inflammatory response, may ultimately feedback and inhibit the antifungal activity of inflammatory cells.

The first signs of clinical disease in our FK mice include the development of corneal opacification and increased tissue thickness at around 48 hours p.i. Both of these pathologies are consistent with corneal edema, which can result as a breakdown in the barrier and water pumping function of the endothelial layer. In addition to aging and various forms of corneal dystrophy (e.g. Fuchs’ dystrophy), contact lens-associated hypoxia is also known to damage endothelial cell function due to a reduction in ATP production and pump efficiency.^70^^,^^71^ We therefore reason that the early development of tissue hypoxia may initiate endothelial dysfunction during FK. As corneal edema slowly progresses, so too will the degree of hypoxia, thus forming a feedforward loop of progressive endothelial decline and worsening disease development. At the later time points, it is likely that accumulating fungal metabolites as well as leukocyte-generated ROS/cytokines further drive endothelial cell death, ultimately leading to a critical loss in endothelial cell integrity and a massive influx of fluid from the anterior chamber.

Although corneal edema is associated with a transient increase in corneal thickness measurement, the end-stage consequence of FK in the clinic is often a critical degradation (thinning) of the stromal matrix that results in perforation and a need for corneal transplantation.^72^^,^^73^ This breakdown in the collagen fibers is likely attributable to secreted proteases from both the fungus as well as matrix metalloproteases (MMPs) secreted by the corneal fibroblasts and infiltrating leukocytes.^6^^,^^74^^–^^77^ Regarding corneal fibroblasts, it has been demonstrated that hypoxic culture increases MMP-1 and MMP-2 production, whereas the production of various collagen species decreases. Indeed, stromal thinning is a consequence of contact-lens driven hypoxia.^78^^,^^79^ Similarly, hypoxia has been shown to increase MMP-9 from neutrophils, along with other collagen damaging molecules, including elastase and myeloperoxidase.^80^ Taken together, the development of hypoxia during FK likely promotes the progression of numerous disease pathologies spanning all cellular layers of the cornea, and the intervention of key cellular regulators of these phenomena (e.g. HIF-1) may play a protective role.

Our studies demonstrate that the development of hypoxia across the epithelium is not only rapid, but strong enough to render the *A. fumigatus* SrbA essential for the establishment of invasive growth into the cornea. This differs somewhat from previous studies in the lungs, whereby the ∆*srbA* mutant is able to initiate growth (germinate) in the airway, but fails to maintain its growth and virulence potential after the onset of neutrophil-driven tissue inflammation at around 2 days p.i. Thus, whereas *A. fumigatus* SrbA is a critical determinant of fungal virulence and disease outcome in both IPA and FK, the stage of infection at which hypoxia develops and blocks infection by *∆srbA* differs between the lung and corneal environments. One key difference may be the high degree of vascularization of the airway, which oxygenates the tissue and buffers the development of hypoxia that would otherwise be driven by the pulmonary epithelial response to fungal antigen.

Azoles are a major class of antifungals for both invasive and ocular *Aspergillus* infection.^81^^–^^86^ These drugs target a key enzyme in the sterol biosynthetic pathway and, in this way, deplete membrane sterol content in the same way hypoxia does. As such, SrbA is activated upon azole treatment and the *∆srbA* mutant is hypersensitive to several drugs in this class, including fluconazole, to which WT *A. fumigatus* is resistant (see Supplementary Fig. S2C).^20^ Thus, pharmacological inhibitors, if they could be developed, of the *A. fumigatus* SrbA pathway would not only be inherently antifungal in hypoxic tissues, but they could potentiate the efficacy of an already available class of antifungals. Ongoing studies in our group are aimed at identifying key regulators of hypoxia adaptation in *Fusarium* species, which, as we have shown here, is likely an important aspect of virulence in this pathogen as well.

In summary, and to our knowledge, this study is the first to demonstrate an important role for corneal hypoxia in the pathobiology of FK. Although our data directly demonstrate an influence of hypoxia on the fungus, it is likely that oxygen depletion will alter the function of effectively all host cells and influence disease pathogenesis. A better understanding of how hypoxia influences the host-fungus interaction, as well as how other FK-relevant pathogens adapt to hypoxia, will undoubtedly give insights into novel therapeutic interventions that improve patient outcomes in patients with FK.

## Supplementary Material

Supplement 1

Supplement 2

Supplement 3
