# Supplementary material for: A spergillus fumigatus Hypoxia Adaptation Is Critical for the Establishment of Fungal Keratitis
Source: Invest Ophthalmol Vis Sci. 2024 Apr 18;65(4):31. doi: 10.1167/iovs.65.4.31 (PMC11044834; doi:10.1167/iovs.65.4.31)
Supplement: Supplement 1 [file iovs-65-4-31_s001.pdf]

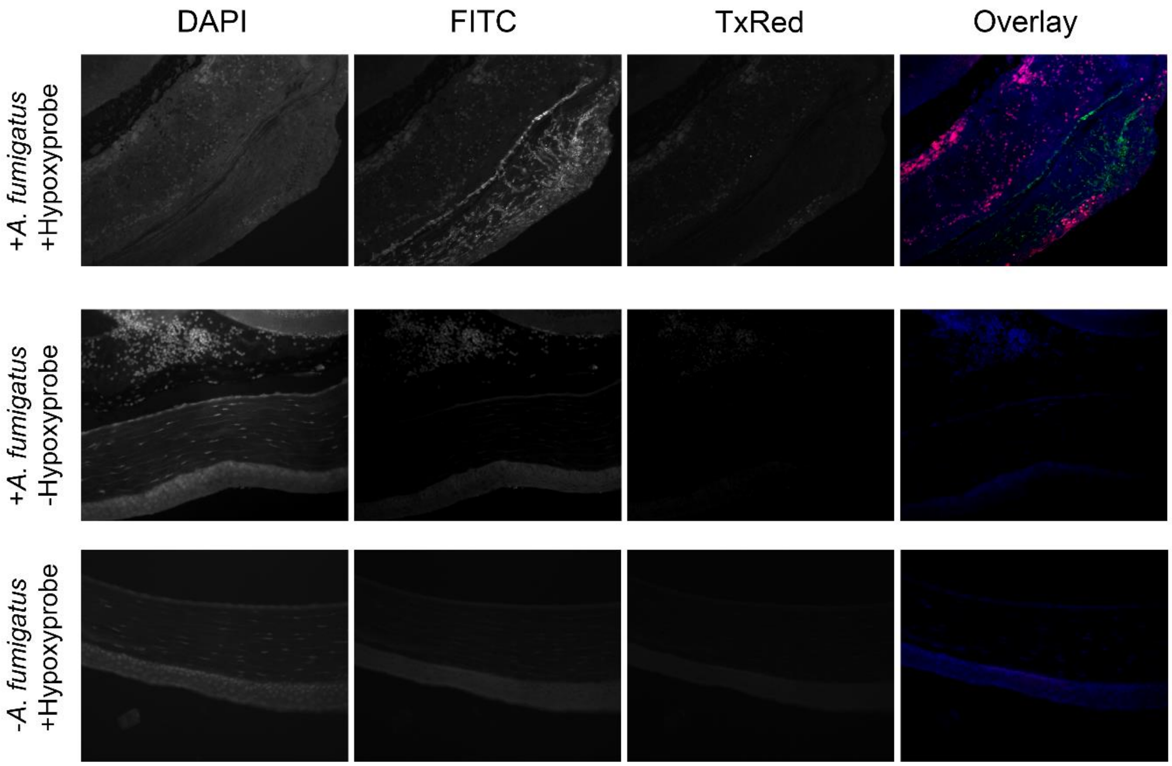

Supplementary Figure 1: Isotype controls for Hypoxyprobe show specific staining. Corneal sections of *A. fumigatus* or sham-inoculated corneas (48 h p.i.) resected from mice injected with Hypoxyprobeor PBS. All sections are stained with DAPI, anti-Aspergillus antibodies (FITC), or anti-pimonidazole antibodies (Dylight 594) and single color channels are shown without alteration to indicate the background levels seen during images. These background levels were removed in the following overlaid image using Metamorph imaging software. Staining shows the host nuclei with DAPI (blue), *A. fumigatus* (green), and Hypoxyprobe (red). All mice in this experiment were male.

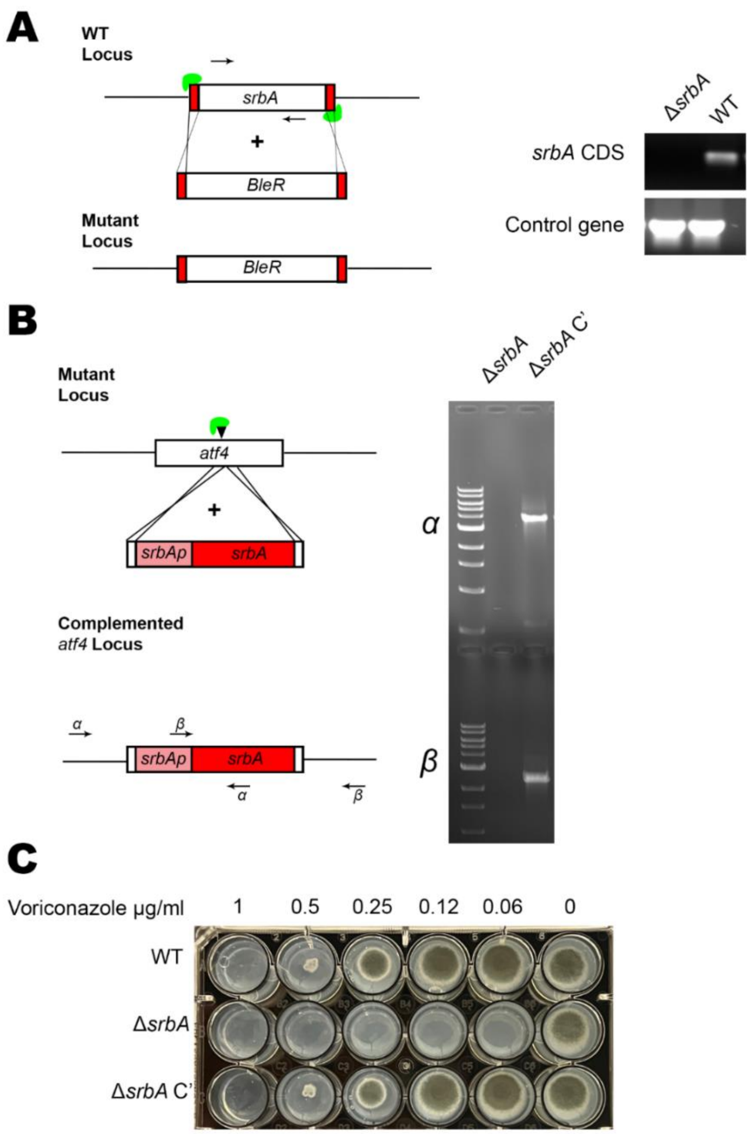

Supplementary Figure 2: Generation of Δ*srbA* mutant and complemented strain (Δ*srbA C'*). (A) An *in vitro* assembled Cas9 RNP approach was used to generate the Δ*srbA* knockout mutant. Mutants were confirmed by PCR (B) The *srbA* gene and its native promoter were amplified with primers containing microhomology sequences to the *atf4* safe haven locus. The *in vitro* Cas9 approach was used to make a single cut in the *atf4* locus and the *srbA* cassette insertion was confirmed by PCR. (C) The Δ*srbA* mutant, the wild-type (WT), and the complemented strain (Δ*srbA* C') were grown at varying concentrations of voriconazole in GMM at 35°C and imaged at 72 h post-inoculation.

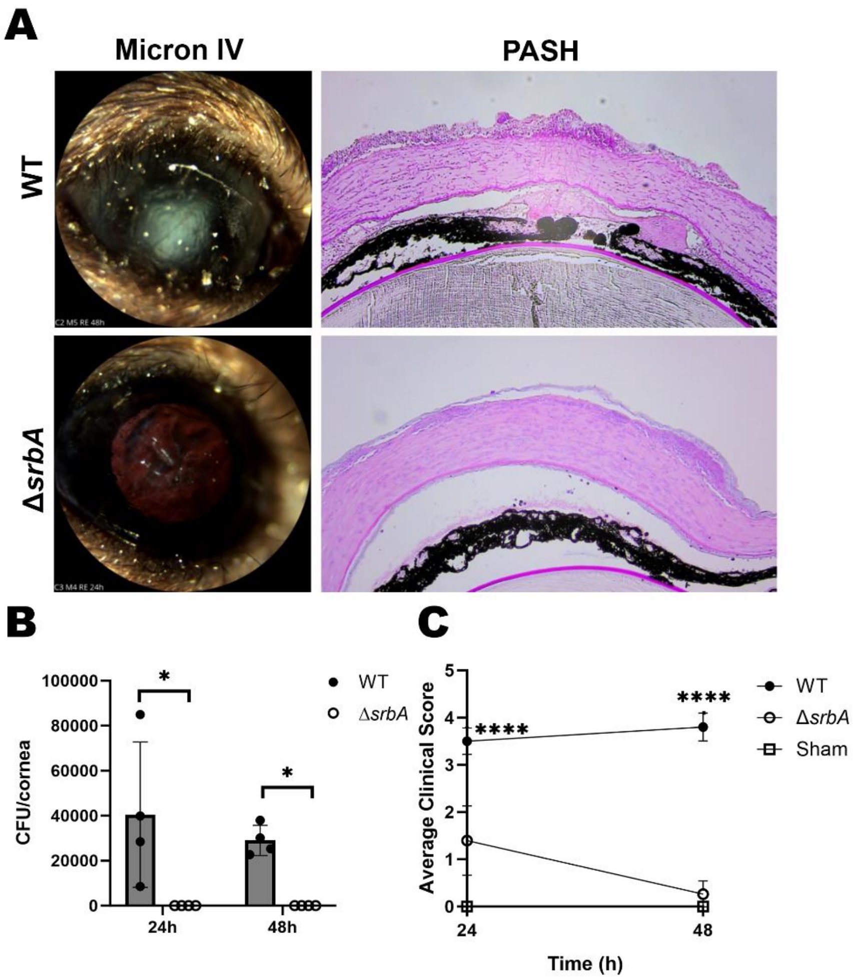

Supplementary Figure 3: Virulence of the ∆*srbA* mutant. Corneas of male C57BL/6J mice were ulcerated and overlaid with inocula of the indicated strains (n=10/group). **(A)** At 24 hours p.i., one animal from each group was euthanized, and their eyes were processed for histology (PASH stained). **(B)** At 24 and 48 hours p.i., animals were euthanized and the corneas were resected and homogenized for colony forming unit quantification (n=4/group for each time point). Groups were compared by the Mann-Whitney U test: *P≤0.05. **(C)** Daily slit lamp images were scored for clinical signs of FK (n=10/group at 24 hours p.i.; n=5/group at 48 hours p.i.). Groups were compared by One-way ANOVA: **** P≤0.0001.
